# Identification of Partner Proteins of the Algae *Klebsormidium nitens* NO Synthases: Toward a Better Understanding of NO Signaling in Eukaryotic Photosynthetic Organisms

**DOI:** 10.3389/fpls.2021.797451

**Published:** 2021-12-22

**Authors:** Pauline Chatelain, Jeremy Astier, David Wendehenne, Claire Rosnoblet, Sylvain Jeandroz

**Affiliations:** Agroécologie, AgroSup Dijon, CNRS, INRAE, Université Bourgogne Franche-Comté, Dijon, France

**Keywords:** nitric oxide synthase, algae, NO signaling, protein partners, interactome

## Abstract

In animals, NO is synthesized from L-arginine by three isoforms of nitric oxide synthase (NOS) enzyme. NO production and effects have also been reported in plants but the identification of its sources, especially the enzymatic ones, remains one of the critical issues in the field. NOS-like activities have been reported, although there are no homologs of mammalian NOS in the land plant genomes sequenced so far. However, several NOS homologs have been found in algal genomes and transcriptomes. A first study has characterized a functional NOS in the chlorophyte *Ostreococcus tauri* and the presence of NOS homologs was later confirmed in a dozen algae. These results raise the questions of the significance of the presence of NOS and their molecular diversity in algae. We hypothesize that comparisons among protein structures of the two KnNOS, together with the identification of their interacting partner proteins, might allow a better understanding of the molecular diversification and functioning of NOS in different physiological contexts and, more generally, new insights into NO signaling in photosynthetic organisms. We recently identified two NOS homologs sequences in the genome of the streptophyte *Klebsormidium nitens*, a model alga in the study of plant adaptation to terrestrial life. The first sequence, named KnNOS1, contains canonical NOS signatures while the second, named KnNOS2, presents a large C-ter extension including a globin domain. In order to identify putative candidates for KnNOSs partner proteins, we draw the protein–protein interaction networks of the three human NOS using the BioGRID database and hypothesized on the biological role of *K. nitens* orthologs. Some of these conserved partners are known to be involved in mammalian NOSs regulation and functioning. In parallel, our methodological strategy for the identification of partner proteins of KnNOS1 and KnNOS2 by *in vitro* pull-down assay is presented.

## Introduction

### Nitric Oxide and Its Biosynthesis

**Figure 1:**
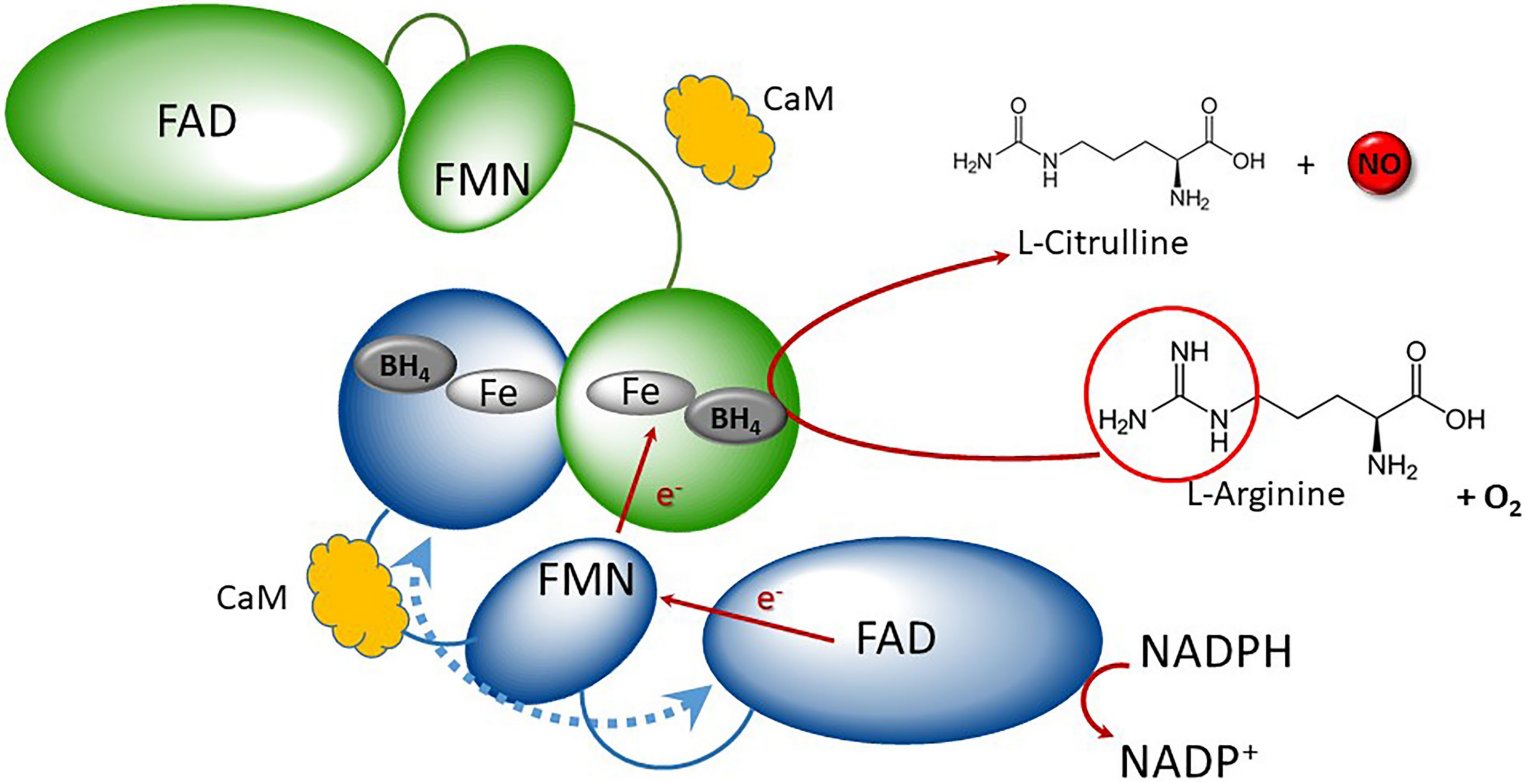
Schematic representation of canonical NOS catalytic mechanism. Canonical NOSs display homodimeric quaternary structure (blue and green monomers). NOS monomers consist of two main domains: the N-terminal oxygenase (round-shaped) and the C-terminal reductase, containing FNR (ellipse) and flavodoxin (small ellipse). They both shelter different redox cofactors: FAD, FMN, heme, and BH4. Reductase and oxygenase domains are interconnected by a Calmodulin (CaM, orange cloud)-binding domain. Electrons are purveyed by NADPH and are shuttled to the flavodoxin *via* the FAD. Upon Cam binding, electron transfer then occurs from the FMN of one monomer (blue) to the heme of the second one (green), resulting to the oxidation of Arginine in presence of oxygen into Citrulline + NO. Adapted from Santolini (2019).

NO is a ubiquitous signaling gaseous molecule which regulates a wide array of physiological, biochemical, and molecular events in animals, plants, and microbes. In mammals, NO is mainly synthetized from L-arginine by three isoforms of nitric oxide synthases (NOSs, EC 1.14.13.39): neuronal NOS (nNOS) or NOS1, inducible NOS (iNOS) or NOS2, and endothelial NOS (eNOS) or NOS3 (Stuehr et al., 2004). Each NOS is a modular enzyme that consists of an N-terminal oxygenase domain and a C-terminal reductase domain, both domains being connected by a short calmodulin binding site (Poulos et al., 1998). In addition to this latter, NOS contains binding sites for NADPH, flavin mononucleotide (FMN), flavin adenine dinucleotide (FAD), tetrahydrobiopterin (BH_4_), and a heme group. Functional NOSs are active as homodimer and transfer electrons from NADPH to their heme center *via* FMN and FAD, where L-arginine is oxidized to L-citrulline and NO (Poulos et al., 1998; Wendehenne et al., 2003). Mechanism for NO catalysis is presented in Figure 1. NOS is present in all kingdoms. In metazoans, phylogenetic and syntenic analyses support the view that NOS was recurrently duplicated in different lineages, acquiring new structural configurations through gains and losses of protein motifs (Andreakis et al., 2011).

In plants, NO production and effects have been reported in many physiological processes even if unresolved issue concerns its enzymatic sources (Besson-Bard et al., 2008; Astier et al., 2018; León and Costa-Broseta, 2020). Indeed, we observe a multiplicity of NO sources, from reductive pathways, to oxidative pathways. Besides nitrite-dependent pathway involving nitrate reductase, NOS-like activities, L-arginine-dependent, and sensitive to mammalian NOS inhibitors, have been reported, although there are no homologs of mammalian NOS in the land plant genomes sequenced so far (Astier et al., 2018).

### NO in Algae

Several reports have suggested that algae from different lineages, such as chlorophytes, charophytes, red algae, or diatoms, could produce NO (Mallick et al., 1999, 2000; Sakihama et al., 2002; Tischner et al., 2004; Chung et al., 2008; Vardi et al., 2008; Chow et al., 2013), and a review of the relevant literature indicates that NO is involved in various functions in algae (Kumar et al., 2015; Astier et al., 2021). Briefly, NO contributes to stress responses, particularly to abiotic ones (e.g., hypoxia, wounding, UV or visible light irradiation, chemicals, heat stress, high concentrations of salt, and metal). NO is also involved in cell physiology (development and metabolism) and could participate in greenhouse gas emission through the reduction of NO into N_2_O in *Chlamydomonas reinhardtii* (Burlacot et al., 2020). As already described in land plants, NO can interact with other signaling molecules, such as hydrogen peroxide and calcium for effective cellular signaling as exemplified in the marine macroalga *Ulva compressa* during the response to copper stress (González et al., 2012).

NOS-like activity has been detected using NOS inhibitors in various marine algae, such as *Ulva compressa* (Chorophytes), *Chattonella marina* (Ochrophyta), or *Symbiodinium microadriaticum* (Dinoflagellates; Kumar et al., 2015), raising the question of the presence of NOS. A first study conducted by Foresi et al. (2010) characterized one functional NOS in the chlorophyte *Ostreococcus tauri* (see also Weisslocker-Schaetzel et al., 2017). In the last decade, massive genome and transcriptome sequencing in plants have revealed several NOS homologs in algae (Jeandroz et al., 2016; Santolini et al., 2017). These NOS are distributed in different phylogenetic groups, in the green lineage but also in the more distant diatoms and dinoflagellates. As described in an exhaustive way by Santolini (2019), the diversity of NOS proteins resides not only in the organismal diversity in which these genes/proteins could be found but also in the number and types of NOSs that are present in any organisms. Indeed, besides the “archetypal” or “standard” NOS, firstly defined in mammals, we now have to consider several different groups of NOSs, corresponding to different architectural types of proteins and perhaps different functions.

Taken together, these results raise questions: why did NOS homologs disappear in land plants during evolution? Why NOS homologs remained in algae and what is the meaning of the NOS molecular diversity in photosynthetic organisms? The characterization and functional analyses of these algal NOSs could provide first answers to these questions.

## Working Hypothesis: No Synthases Interact with Partner Proteins That Could Modulate Their Activities and Specificities

Protein–protein interactions (PPI) are the crucial events in cellular signaling mechanisms. In mammals, the three identified NOS isoforms display specific profiles of expression, cellular/subcellular localization, regulation, or catalytic properties. Emerging evidence indicates a role for PPI as a regulator of NOS activity and therefore specificity. For instance, destabilization of the active NOS dimeric structure or preventing its dimerization by PPI leads to its inactivation (Zhou and Zhu, 2009; Cinelli et al., 2020) while NOS activity (NO production) can be optimized by a partner protein which facilitates the delivery of its substrate (Kone, 2000). Moreover, specific interaction of the PDZ N-ter domain with the dystrophin complex is responsible for human NOS1 subcellular distribution (Brenman et al., 1995). PPI are also at the origin of multiple component signaling modules, associating NOS, scaffolding, and S-nitrosated protein. For instance, the NOS2 interaction with the protein PSD95, leading to the regulation of the NMDA receptor by S-nitrosylation, constitutes a noteworthy illustration of the spatiotemporal aspect of NO signaling (Stamler et al., 2001).

We hypothesize that comparisons among protein structures of the NOS, together with the identification of their partner proteins, might allow a better understanding of the molecular diversification and functioning of NOS in different physiological contexts and, more generally, new insights into NO signaling in photosynthetic organisms.

In order to test this hypothesis, we use the charophyte (streptophytes) *Klebsormidium nitens* as a biological model to investigate these NOSs and their structural and functional diversity. *K. nitens* presents two interests. Firstly, its complete genome sequence has revealed several keys signaling modules, known to interact with NO in land plants (e.g., phytohormones) and required for plant terrestrial adaptation (Hori et al., 2014). Secondly, BLAST searches using OtNOS revealed the presence of two protein sequences showing specific NOS molecular patterns but different architectures (domain organization), which makes it possible to address the question of the structure/activity relationships of NOS.

## Molecular Diversity of Knno Synthases and Their Relatives in Algae

### Two Putative KnNOSs Are Found in the *K. nitens* Genome

#### KnNOS1, The “Standard” Model

**Figure 2:**
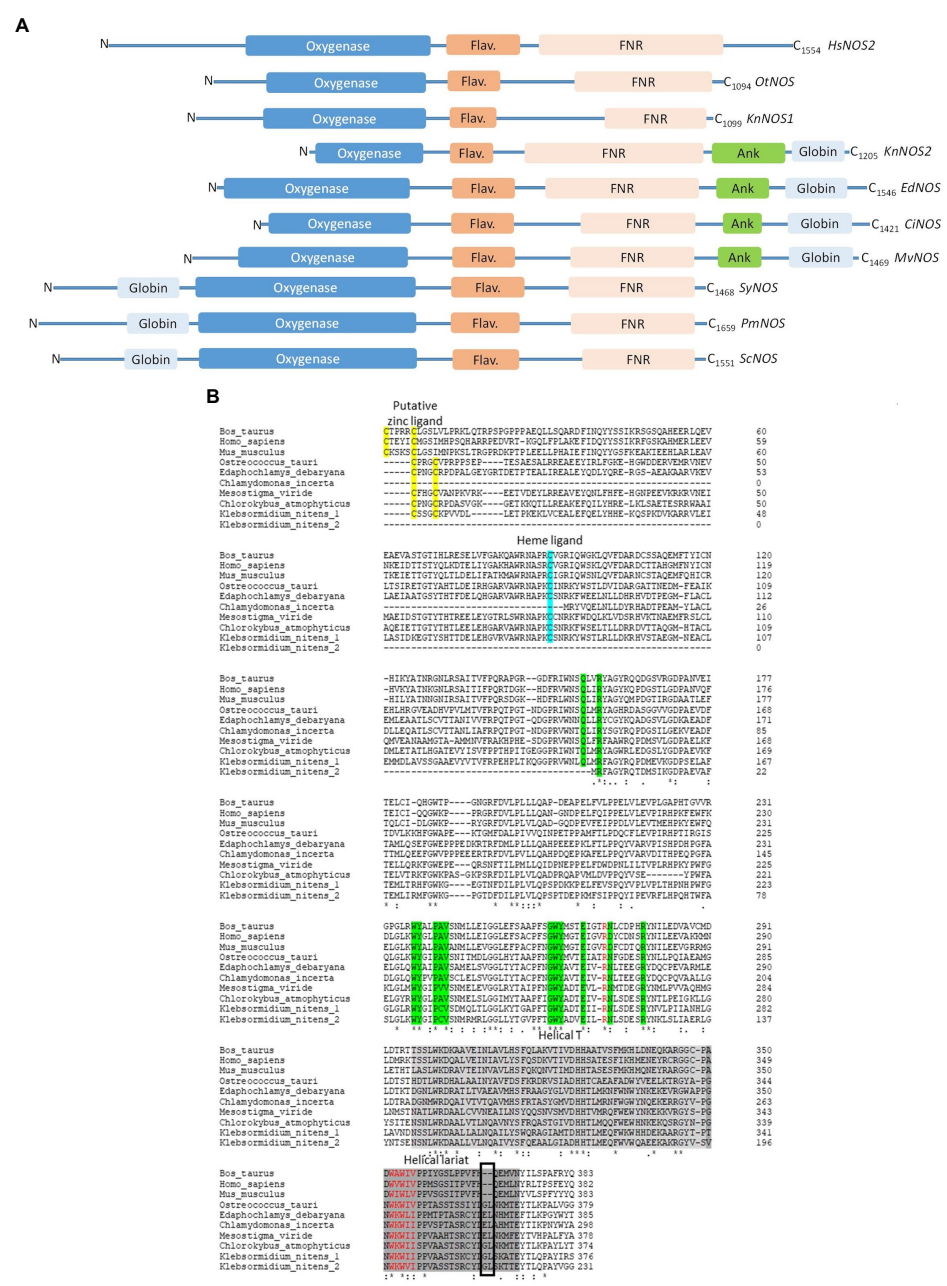
**(A)** Schematic representation of primary sequences of NOS-like proteins from *Homo sapiens* (*HsNOS2*, AAB60654), *Ostreococcus tauri* (*OtNOS,* OUS45267), *Klebsormidium nitens* (*KnNOS1,* KFL_000760350 at the top and *KnNOS2,* KFL_006460015 at the bottom), *Edaphochlamys debaryana* (*EdNOS,* KAG2495438), *Chlamydomonas incerta* (*CiNOS,* KAG2438015), *Mesostigma viride* (*MvNOS,* Mv7497-RA-2, Joint Genome Institute PhytoCosm), *Synechococcus* sp. (*SyNOS,* WP_006458277), *Pseudo-nitzschia multistriata* (PmNOS), and *Skeletonema costatum* (ScNOS). Three NOSs share a common oxygenase domain (blue). The FMN domain is represented by a dark orange flavodoxin module (Flav.) and the FAD/NADPH domain is represented by a light orange FNR box. Additional domains comprise a globin domain (light blue box), an ankyrin-repeat domain (Ank, green). Conserved domains have been identified thanks to NCBI and domains alignment analyzed through Clustal Omega. **(B)** Comparative analysis of NOS oxygenase domains from *Bos Taurus* (AAA30669.1), *Homo sapiens* (*HsNOS2*, AAB60654), *Mus musculus* (AAA39834.1), *Ostreococcus tauri* (OUS45267), *Edaphochlamys debaryana* (KAG2495438), *Chlamydomonas incerta* (KAG2438015), *Mesostigma viride* (*MvNOS,* Mv7497-RA-2, Joint Genome Institute PhytoCosm), *Chlorokybus atmophyticus* (*CaNOS*, chrsp63S00556, Joint Genome Institute PhytoCosm), and *K. nitens* (1, KFL_000760350 and 2, KFL_006460015). Arginine binding residues are colored in green. The black box highlights the insert observed in the helical lariat of non-mammalian NOSs. Alignment has been performed through Clustal Omega.

This protein of 1099 aa (GAQ81306.1) shares 36% of identity with OtNOS, the first NOS characterized in algae (Foresi et al., 2010). KnNOS1 possesses classical mammalian NOS architecture consisting of oxygenase and reductase domains (Figure 2A). Globally, KnNOS1 structure is close to the other NOSs described in green algae (Santolini et al., 2017). Compared to mammalian NOSs, these algal NOSs lack the key conserved residues of the N-ter hook as well as those involved in binding the dihydroxypropyl side chain of BH_4_. A structural analysis of the NOSoxy domain shows that algal NOSs conserve the key structural features of mammalian, including the proximal heme ligand Cys, the L-Arg- and BH_4_-binding residues, and a modified helical lariat and helical T region that are involved in binding the pterin cofactor and in the interactions between monomers that help stabilize the homodimer (Figure 2B). They also possess an atypical putative zinc-binding region (Cys-X3-Cys instead of the typical Cys-X4-Cys; Santolini et al., 2017).

#### KnNOS2, The “NOS-Globin” Model

**Figure 3:**
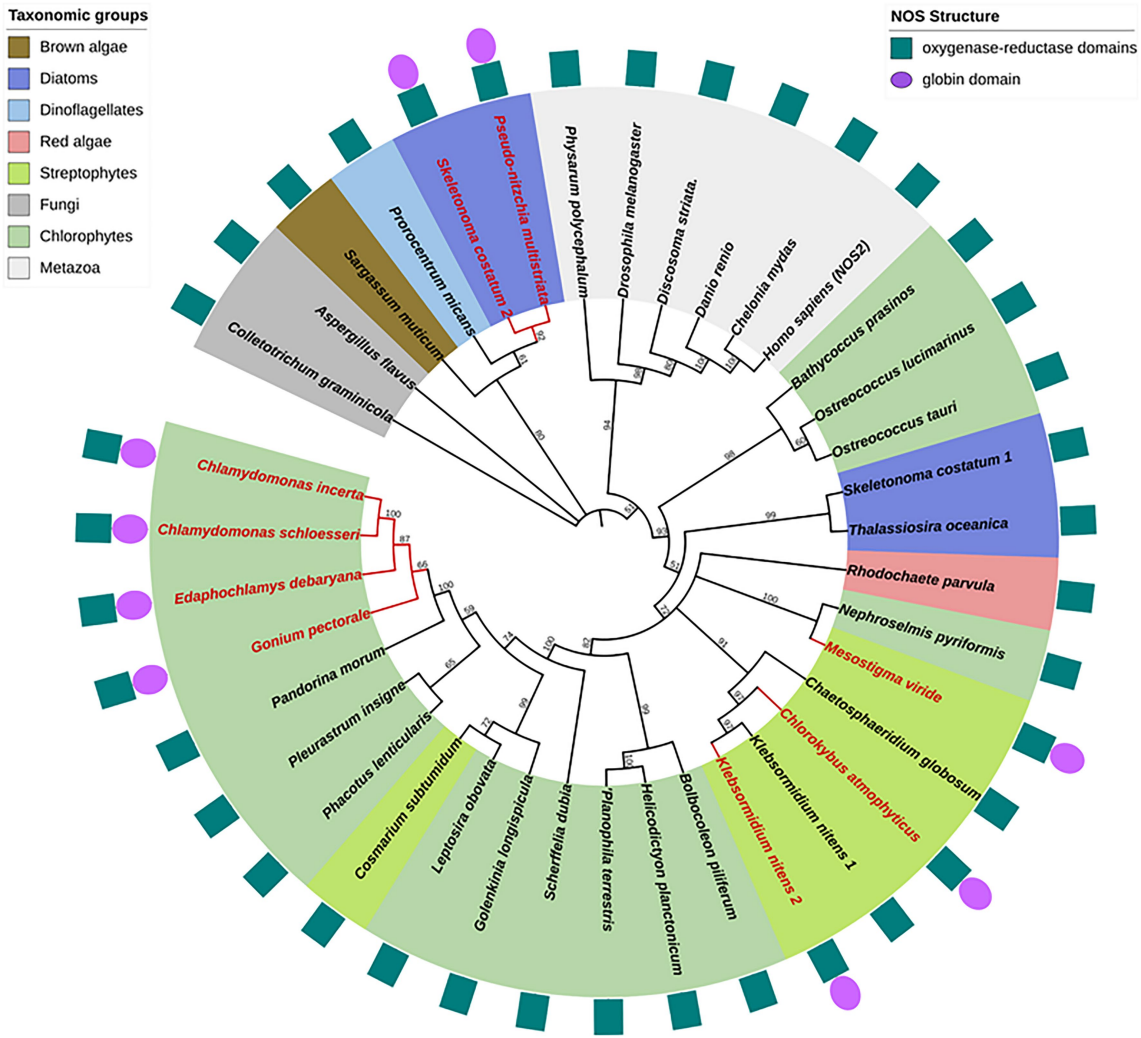
Phylogenetic reconstruction of NOS proteins. Algal NOS sequences were extracted from databases (see Santolini et al., 2017). NOS proteins were aligned with ClustalW and the phylogenetic tree was constructed by maximum likelihood method using MEGA (Tamura et al., 2021). Bootstrap analysis was carried out using 500 replicates. All nodes with bootstrap values less than 50% were collapsed into polytomies. Branches and labels in red indicate chimeric structures. Protein domain organization is depicted around the phylogenetic tree.

we identified in the *K. nitens* genome a second putative NOS sequence showing a large C-ter extension of approximately 380 residues (GAQ90485.1). Conserved protein domain analysis using CD-Search in NCBI (Marchler-Bauer et al., 2011) reveals an ANK motif and a globin domain. The globin domain belongs to the M family globin domain and shows a characteristic α-helical secondary structure with two distal and proximal His residues involved in the coordination of heme-Fe (Vázquez-Limón et al., 2012). The combination of a globin domain to a NOS protein in the C-ter end has already been identified by Santolini (2019) in the green algae *Gonium pectorale* (Chlorophytes; Figure 3). Interestingly, we recently identified other NOS proteins showing the chimeric NOS-Globin organization in two other charophytes *(Chlorokybus atmophyticus* and *Mesostigma viride*) and three chlorophytes *Chlamydomonas incerta, C. schloessori* et *Edaphochlamys debaryana* (Figures 2, 3). This NOS-Globin organization seems to be evolutionary significant as it appears in three of the six major clades of charophytes, the earliest diverging lineages of extant streptophytes. Biological functions of these proteins need to be studied to validate their role in algal physiology. The association of globin and NOS domains has been firstly characterized in the free-living marine unicellular cyanobacterium *Synechococcus* PCC 7335 (Correa-Aragunde et al., 2018), but in this protein, the globin domain is located at the N-ter of the protein (Globin-NOS). Biochemical analysis showed that SyNOS produces NO and NO_3_^−^ *in vitro* and data suggest that the globin domain is mainly responsible for NO_3_^−^ production (Picciano and Crane, 2019). It is proposed that the globin domain in SyNOS could confer new functional capabilities for N metabolism (Correa-Aragunde et al., 2018). Interestingly, chimeric globin-NOSs similar to SyNOS appear in some diatom genomes, such as *Pseudo-nitzschia multistriata* (PmNOS) and *Skeletonema costatum* (ScNOS; Di Dato et al., 2015; Figure 2A).

### Phylogenetic Diversity of NO Synthases in Photosynthetic Organisms

As previously reported, algal NOSs are unequally distributed in the photosynthetic organism phylogeny (Jeandroz et al., 2016; Santolini et al., 2017). They were found in the green lineage (streptophytes and chlorophytes) but also in red algae, diatoms, dinoflagellates, and brown algae (Figure 3). Several discrepancies between the standard plant phylogeny and the one deduced from NOS proteins are noticed, indicating duplication/loss events or horizontal gene transfers along NOS gene evolution and consequently putative functional homoplasy. For examples, chlorophytes NOSs are not grouped together and the green lineage (streptophytes and chlorophytes) is not, contrary to plant phylogeny, a monophyletic group since several diatoms and red algae NOS belong to the same clade. Interestingly, both KnNOSs are grouped together, and KnNOS2 is not related to other NOS-Globin found in *E. debaryana* and in the two *Chlamydomonas* species. This indicates that the NOS-globin chimeric organization, found in seven species, could have independent origins. The Globin-NOS organization, firstly characterized in the cyanobacterium *Synechococcus* PCC 7335, is restricted to the two diatoms *P. multistriata* and *S. costatum*. Noticeably, this latter species harbors a second NOS, with a classical organization, which is distantly related from the NOS-Globin in the phylogenetic tree.

## Putative No Signaling Actors In *K. nitens* Genome Sequence

In mammals, the biological effects of NO are mediated notably *via* a NO/cGMP depending cascade which requires the mobilization of soluble guanylyl cyclases (sGCs), cGMP, cGMP-dependent protein kinases (PKGs), and cyclic nucleotide phosphodiesterases (PDEs; Martínez-Ruiz et al., 2011). We recently investigated the presence of these major NO-signaling cascade components in the green lineage (Astier et al., 2019). None of the +1000 species tested harbored all the required enzymes necessary for the classical NO/cGMP depending cascade. However, a few homologs of soluble guanylate cyclases, cGMP-dependent protein kinases, cyclic nucleotide-gated channels, and cGMP-regulated PDEs were identified punctually in some of the organisms tested, mainly algae.

Interestingly, among the candidates identified, some were further characterized, such as the sGCs CYG12, CYG56, and CYG11 from *C. reinhardtii*. CYG12 protein shares 40 to 50% identity with animal GCs and an initial structural analysis demonstrated that its conformation allows a functional cyclase activity (Winger et al., 2008). Recently, the enzyme was demonstrated to be functional and to participate in hypoxia responses in *C. reinhardtii* (Düner et al., 2018). Interestingly, NO is also involved in hypoxia responses (Gupta et al., 2020; Manrique-Gil et al., 2021). However, *in vitro* approaches using NO donors on the recombinant protein failed to demonstrate an increase of the enzymatic activity (Düner et al., 2018). Therefore, *in vivo* approaches are required to further confirm or invalidate the involvement of CYG12 in a NO/cGMP signaling cascade. Another guanylate cyclase, CYG56, was found to be involved in the regulation of nitrate assimilation, through its participation in the NO/cGMP-dependent signaling cascade mediating nitrate reductase repression induced by ammonium (de Montaigu et al., 2010). However, similarly to CYG12, CYG56 cyclase activity did not show NO sensitivity *in vitro*. Concerning CYG11, the protein displays unique features as compared to other described eukaryotic guanylate cyclases (Horst et al., 2019). Structurally, this enzyme possesses two HNOX domains and is active as a homodimer. Interestingly, its activity is increased upon carbon monoxide (CO) exposure, rather than NO exposure. Based on its enzymatic parameters, CO may represent the physiological ligand of CYG11, making this enzyme a CO-sensing protein in *C. reinhardtii,* although the confirmation of this hypothesis will require further investigations. The characterization of these three guanylate cyclases demonstrates the importance of cGMP-dependent signaling in algae, although a direct link with NO-dependent signaling remains to be clearly established.

The first proper biochemical characterization of a plant cGMP/cAMP-dependent protein kinase was only reported recently in rice (Shen et al., 2019). Presenting atypical features compared to animal ones, notably the coexistence of a phosphatase and a kinase domain within the sequence, this PKG was found to be involved in gibberellin (GA) signaling and salt stress responses, two pathways where NO acts as a signaling molecule. Nevertheless, the corresponding molecular actors, particularly those related to NO and cGMP synthesis, remain to be identified to confirm the existence of a GA/NO/cGMP signaling cascade in rice. Interestingly, homologs of this rice PKG are generally present in single copy and well distributed among the green lineage, in algae and land plants. Concerning the cGMP turnover, the presence of PDEs related to NO signaling in the green lineage is yet to be demonstrated. A unique description of a putative class I PDE has been reported in *C. reinhardtii* (Gross and Durner, 2016), but its activity is yet to be characterized. Additionally, the first functional cGMP-activated PDE from plants was recently described in *Arabidopsis thaliana* (Isner et al., 2019). However, here again, no information about the NO-dependency of this protein activity, related to UV-A responses, has been reported.

Similarly, the relation between NO and CNGCs is not fully elucidated in plants. Out of our bioinformatic analyses, no NO-signaling-related CNGC homologs were found in the green lineage (Astier et al., 2019). Accordingly, no regulation of plant or algae CNGCs by NO-dependent mechanisms has been reported so far.

A closer search on the putative NO-signaling modules that can be found in *K. nitens* revealed the presence of some candidates. If no homologs of animal type NO-dependent sGC of CNGCs could be retrieved, the search for protein homologs of the putative CrPDE (XP_001689825.1) and rice PKG (Q6K3D4_ORYSJ), with a minimum of 30% identity on 60% of the query length, leads to the identification of, respectively, 3 (GAQ77586.1, GAQ86043.1, and GAQ84593.1) and 1 (GAQ83023.1) candidates. The determination of their respective roles in *K. nitens* together with their potential link with NO signaling and KnNOSs activities constitutes a promising ax of investigation.

## Identification and Role of Noss Interacting Partner Proteins

**Figure 4:**
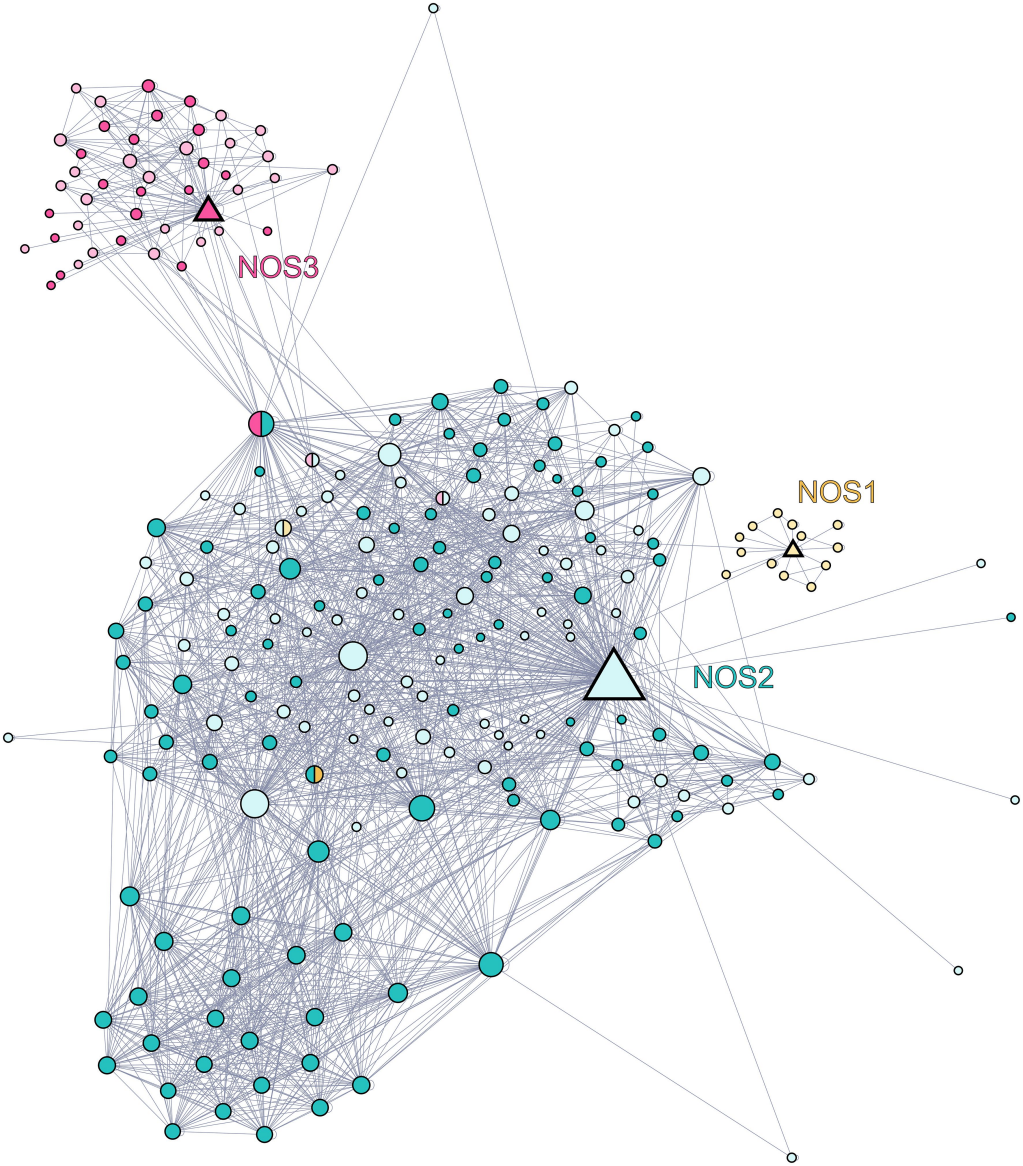
Network of the protein–protein interaction network of human NOS1 (neuronal NOS; P29475), NOS2 (inducible NOS; P35228), and NOS3 (endothelial NOS; P29474). Data were downloaded from the BioGRID database and graphical representation was carried out using Gephi software. Each node represents a protein, and the size of the nodes is proportional to the number of connections with other nodes. The links between nodes represent physical interactions between proteins. The triangle-shaped nodes represent the three NOSs, yellow for NOS1, blue for NOS2, and red for NOS3. The circles-shaped nodes represented in blue are partners of NOS2, those in red are partners of NOS3, and those in yellow are partners of NOS1. Nodes represented in pastel shade do not have orthologs in *K. nitens* while those in dark colors are conserved in *K. nitens*.

In order to identify putative candidates for putative KnNOSs partner proteins, we first draw the PPI network of the three human NOS isoforms using the BioGRID database, which congregates PPI data based on experimental approaches (Figure 4). This network reconstruction indicates specific interactions between each of the three isoforms with privileged partners. NOS2 presents the largest numbers of interactors with 175 nodes, compared to NOS1 and NOS3 with 16 and 46 nodes, respectively. Some partners interact with more than one human NOS and allow the interconnection of the three subnetworks. Each of the three NOS interacts with itself, as suggested by the active dimeric form of NOS (Venema et al., 1997).

Starting from this confirmed NOS interacting protein list identified in humans, we then look for the presence of their orthologs in *K. nitens*. We developed an *in silico* approach using OrthoFinder, a powerful tool which allows whole-genome comparisons (Emms and Kelly, 2019). Among partner proteins of human NOS1, NOS2, and NOS3, 6.25, 58.3, and 45.8% are conserved in the proteome of *K. nitens*, respectively. These conserved proteins are represented in dark shades in PPI network (Figure 4). Since orthologous proteins are more likely to share the same function, we are able to hypothesize on the biological role of *K. nitens* orthologs (Emms and Kelly, 2015). Some of these conserved partners are known to be involved in mammalian NOSs regulation and functioning. Several interesting candidates were identified and detailed below.

### Control of NOS Activity: The Role of HSP90 and Actin

NOS activity can be enhanced by formation of a ternary complex with HSP90 and globular β-actin (Table 1). Firstly, HSP90 binds to NOS which results in an increase in NOS activity as well as an increase in affinity for β-actin. Secondly, binding of β-actin results in an increase of HSP90 degradation, and in the dissociation of the ternary complex, thus limiting the time in which NOS3 is active. This mechanism increases the production of NO when the globular form of β-actin is favored over the fibrillar form while maintaining a negative feedback (Ji et al., 2007).

**Table 1:** List of human NOSs partners identified from the BioGRID database and their orthologs in *Klebsormidium nitens* genome.

Hs protein		Ortholog in Kn		Interaction with		Action on HsNOSs
Name	UniProtKB accession	Name	UniProtKB accession			
HSP90	P07900	HSP	A0A1Y1HY34	NOS2/NOS3	Increase activity
		HSP	A0A1Y1HT80		
		HtpG	A0A1Y1HL34		
β-actin	P60709	Actin	A0A1Y1IK53	NOS3	Negative feedback
STUB1	Q9UNE7	STUB1	A0A0U9HIG1	NOS1/NOS2	Proteasomal degradation
Elongin C	Q15369	Elongin C	A0A1Y1IDM0	NOS2	Proteasomal degradation
ADRM1	Q16186	Rpn13	A0A1Y1HR80	NOS2	Proteasomal degradation
UCH37	Q9Y5K5	UCH	A0A1Y1HNS5	NOS2	Proteasomal degradation
Rac2	P15153	Ras	A0A1Y1IHY9	NOS2	Cellular distribution
NOSIP	Q9Y314	RING	A0A1Y1HYP3	NOS3	Cellular distribution

### Proteasomal Degradation of NOS: The Role of STUB, Elongin C, ADRM1, and UCH37

Binding of partners with NOS could trigger the proteasomal degradation of the relevant NOS isoform and could constitute another level of regulation (Table 1). For example, in human cells, it has been shown that STUB1, *via* its ubiquitin ligase activity triggers the degradation of NOS1 by ubiquitinating the calmodulin binding site (Clapp et al., 2012). A similar mechanism has been described for Elongin C which interacts and ubiquitinylates NOS2. This ubiquitin motif is then recognized by the 19S proteasome subunit ADRM1, followed by the recruitment of UCH37, leading to proteasomal degradation of NOS2 (Mazumdar et al., 2010; Nishiya et al., 2011).

### Changes of NOSs Intracellular Distribution: The Role of Rac2 and NOSIP

Isoform-specific regulators of NOS intracellular distribution have been identified (Table 1). In the case of NOS2, interaction with Rac2 during inflammatory response leads to an increase of NOS activity through spatial redistribution within the cell (Kuncewicz et al., 2001). In the case of NOS3, the binding of NOSIP in C-terminal domain negatively modulates NOS activity by uncoupling NOS3 from plasma membrane caveolae (Dedio et al., 2001).

Thereby, our preliminary *in silico* approach highlights the promising presence of two NOSs and putative numerous NOS interacting partners in the genome of *K. nitens*. We hypothesize that these enzymes would participate in a complex signaling networks, as the ones described in animals, and we seek to determine its specificities and roles.

### Identification and Functional Analysis of KnNOS Partner Proteins

**Figure 5:**
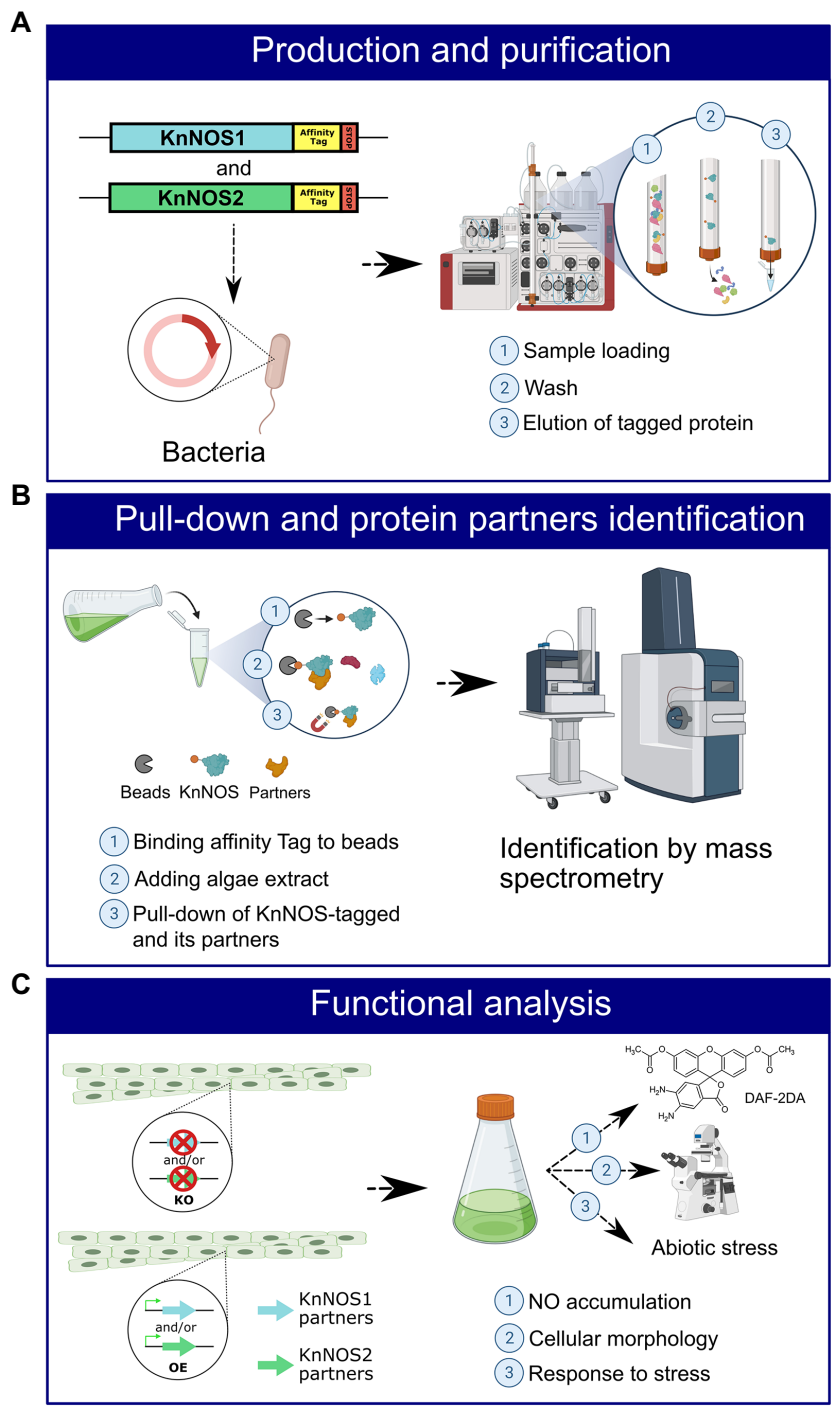
Schematic representation of the strategy for identifying NOSs protein partners and their characterization. **(A)** An affinity tag will be added by molecular engineering to the sequence coding for KnNOS1 and KnNOS2. The two inserts will be cloned in vector, and the two recombinant proteins will be produced in heterologous system. Recombinant proteins KnNOS1 and KnNOS2 will be purified by affinity chromatography. **(B)** Purified KnNOS1 and KnNOS2 will be coated on magnetic beads. Pull-down assay will be carried out to precipitate KnNOSs and their respective partner proteins. Partner proteins will be identified by mass spectrometry. **(C)** A functional analysis using targeted genetic approaches will allow the characterization of the physiological functions of KnNOSs through study of their partner proteins.

Our methodological strategy for the identification of partner proteins is presented in Figure 5.

The first step will be to produce KnNOS1 and KnNOS2 proteins in heterologous bacterial system (Figure 5A). Once the two tagged recombinant proteins produced by bacteria, purified and then fixed on beads by affinity for the tag, *in vitro* pull-downs will be carried out through incubations with native extracts of algae proteins. Partners pulled-down through tagged KnNOSs will be then identified by nano-liquid chromatography tandem mass spectrometry analysis (Figure 5B). Negative control will be performed using protein extracts incubated with the tag alone fixed on beads. The list of identified protein partners will be compared to the one obtained with the *in silico* analysis in order to choose the most promising candidates.

The second step will be a functional analysis of the protein partners. It will consist in genetic approaches implying the generation of knock-out and overexpressing *K. nitens* mutant lines for several candidate partners (Figure 5C). Different methods of transformation could be tested. The electroporation method is operational in several algal strains and particularly in charophytes (Kawai et al., 2021). If unsuccessful, we will test an *Agrobacterium tumefaciens*-based approach. This latter was efficiently adapted for transformation of microalgae (Doron et al., 2016). The functional consequences of these mutations will be based on the analysis of (1) NO production by fluorimetry with DAF-2DA, (2) cellular morphology by microscopy, and (3) algae responses to abiotic stress.

## Conclusion

The discovery of two NOS isoforms in the *K. nitens* genome confirms the already observed molecular diversity of these proteins, illustrated by intra-domain amino-acid polymorphisms as well as recombination of protein domains. This diversity reflects the complex evolution of NO signaling in photosynthetic organisms. A first analysis, conducted *in silico*, has confirmed the presence of putative partner proteins of NOS in the *K. nitens* genome. Now, the use of *in vitro* assays followed by functional characterization of candidate proteins, in different physiological contexts, will certainly help us to improve our understanding on NOSs regulation and their roles in algae.

## Data Availability Statement

The original contributions presented in the study are included in the article/supplementary material, and further inquiries can be directed to the corresponding author.

## Author Contributions

PC, JA, and SJ conducted the *in silico* analyses. SJ, CR, and DW conceived the study. All authors wrote and edited the manuscript.

## Funding

Work in our lab is currently supported by the Ministère de l’Enseignement supérieur, de la Recherche et de l’Innovation, Investissements d’Avenir program, project ISITE-BFC NOISELESS (contract ANR-15-IDEX-0003; grant NOISELESS-RA18041.AEC.IS) and the Agence Nationale de la Recherche, project ALGAE-NOS (grant ANR-18-CE20-0022-02).

## Conflict of Interest

The authors declare that the research was conducted in the absence of any commercial or financial relationships that could be construed as a potential conflict of interest.

## Publisher’s Note

All claims expressed in this article are solely those of the authors and do not necessarily represent those of their affiliated organizations, or those of the publisher, the editors and the reviewers. Any product that may be evaluated in this article, or claim that may be made by its manufacturer, is not guaranteed or endorsed by the publisher.
